# Engineering *Lactococcus lactis* for Increased Vitamin K2 Production

**DOI:** 10.3389/fbioe.2020.00191

**Published:** 2020-03-18

**Authors:** Cathrine Arnason Bøe, Helge Holo

**Affiliations:** ^1^Laboratory of Microbial Gene Technology, Faculty of Chemistry, Biotechnology and Food Science, Norwegian University of Life Sciences, Ås, Norway; ^2^Tine SA, Oslo, Norway

**Keywords:** *Lactococcus lactis*, menaquinone, vitamin K2, mevalonate kinase, prenyl diphosphate synthase, MK-8, MK-9, MK-3

## Abstract

Cheese produced with *Lactococcus lactis* is the main source of vitamin K2 in the Western diet. Subclinical vitamin K2 deficiency is common, calling for foods with enhanced vitamin K2 content. In this study we describe analyses of vitamin K2 (menaquinone) production in the lactic acid bacterium *L. lactis* ssp. *cremoris* strain MG1363. By cloning and expression from strong promoters we have identified genes and bottlenecks in the biosynthetic pathways leading to the long-chained menaquinones, MK-8 and MK-9. Key genes of the biosynthetic menaquinone pathway were overexpressed, singly or combined, to examine how vitamin K2 production can be enhanced. We observed that the production of the long menaquinone polyprenyl side chain, rather than production of the napthoate ring (1,4-dihydroxy-2-naphtoic acid), limits total menaquinone synthesis. Overexpression of genes causing increased ring formation (*menF* and *menA*) led to overproduction of short chained MK-3, while overexpression of other key genes (*mvk* and *llmg_0196*) resulted in enhanced full-length MK-9 production. Of two putatively annotated prenyl diphosphate synthases we pinpoint *llmg_0196* (*preA*) to be important for menaquinone production in *L. lactis*. The genes *mvk, preA, menF*, and *menA* were found to be important contributors to menaquinone levels as single overexpression of these genes double and more than triple the total menaquinone content in culture. Combined overexpression of *mvk, preA*, and *menA* increased menaquinone levels to a higher level than obtained individually. When the overproducing strains were applied for milk fermentations vitamin K2 content was effectively increased 3-fold compared to the wild type. The results provide a foundation for development of strains to ferment foods with increased functional value i.e., higher vitamin K2 content.

## Materials and Methods

### Bacterial Strains, Plasmids, and Culture Conditions

Strains and plasmids used in this study are listed in Table 1. *Lactococcus lactis* ssp. *cremoris* MG1363 or NZ9000 were used as hosts for expression studies. *E. coli* NEB10 beta (NEB, Ipswich, MA, USA) electrocompetent cells were used for routine cloning and One Shot™ Mach1™ T1R (Invitrogen, Carlsbad, CA, USA) chemically competent cells were used for subcloning. Unless otherwise stated lactococci were inoculated into M17 medium (Formedium, Norfolk, UK) supplemented with 0.5% glucose and antibiotics required for selection (erythromycin 10 μg/ml, tetracyclin 12.5 μg/ml, chloramphenicol 10 μg/ml), and grown over night at 30°C under static conditions. Nisin (Sigma, St. Louis, MO, USA) was added from a 1 mg/ml (w/v) stock in 0.05% (v/v) acetic acid when the OD600 in the cultures reached 0.2 to induce expression from the *nisA* promoter. For milk fermentations 10% dry skimmed milk supplemented with 0.5% (w/v) glucose and 1% (w/v) tryptone was heat sterilized at 90°C for 45 min. Tubes with five ml milk was inoculated from a GM17 preculture and fermented for 20 h at 30°C. Nisin was added 1 h after start of the milk fermentations at a concentration of 2 ng/ml.

**Table 1:** Strains and plasmids used in this study.

**Strain or plasmid**	**Relevant characteristic(s)**	**Reference or source**
**Strains**		
*L. lactis*		
MG1363	*Lactococcus lactis* ssp. *cremoris*	Gasson, 1983
NZ9000	MG1363; *pepN*::*nisRK*	Kuipers et al., 1998
*E. coli*		
NEB 10 beta	Commercial cloning host	NEB
One Shot™ Mach1™- T1R	Commercial cloning host used for pCR™Blunt II	Invitrogen
**Plasmids**		
pMG36e	Em^r^, *L.lactis* expression vector, P32 promoter	van de Guchte et al., 1989
pMenF	pMG36e constitutively expressing *menF*	This study
pMenA	pMG36e constitutively expressing *menA*	This study
pHmcM	pMG36e constitutively expressing *hmcM*	This study
pThiL-MvaA	pMG36e constitutively expressing *thiL* and *mvaA*	This study
pMvk	pMG36e constitutively expressing *mvk*	This study
pMvaD-Pmk	pMG36e constitutively expressing *mvaD* and *pmk*	This study
pFni	pMG36e constitutively expressing *fni*	This study
pIspA	pMG36e constitutively expressing *ispA*	This study
pAS-222	Tet^r^, *L. lactis* vector, derivate of pG^+^host4 and pBluescript SK	Jonsson et al., 2009
pCR™Blunt II	Kan^r^, Zeo^r^, TOPO vector for subcloning in *E. coli*, derivative of pUC	Invitrogen
pNZ8037	Cm^r^, inducible expression vector, P*_*nisA*_* promoter	de Ruyter et al., 1996
pPreA	pNZ8037 expressing *preA* upon induction with nisin	This study
pGerCA-IspB	pNZ8037 expressing *gerCA* and *ispB* upon induction with nisin	This study
pPreA-MenA	pNZ8037 expressing *preA* and *menA* upon induction with nisin	This study
pSMART	Amp^r^, cloning vector, low copy, derivative of pUC	Lucigen
pIL252	Em^r^, *L. lactis* low copy vector, derivative of pAMb1	Simon and Chopin, 1988
pHH145	Em^r^ Amp^r^, pSMART, and pIL252 combined, high DNA capacity shuttle vector for *E. coli* and *L.lactis*	This study
pCTR	pHH145 constitutively expressing *tetM* from P32	This study
pMEV-PP	pHH145 constitutively expressing *hmcM, thiL, mvaA, mvk, preA, mvaD, pmk, fni, ispA* and *tetM* from P32	This study
pMEV-PP-2	pHH145 constitutively expressing *hmcM, thiL, mvaA, mvk, mvaD, pmk, fni, ispA*, and *tetM* from P32	This study

### Construction of Plasmids

A shuttle vector, pHH145, with capacity to carry large DNA fragments in *L. lactis* was made by ligating EcoRI restricted pSMART and the high DNA capacity vector for Gram positive bacteria, pIL252 (Simon and Chopin, 1988) and propagated in *E. coli* (Supplementary Figure 2).

All genes were amplified from MG1363 chromosomal DNA except the tetracycline resistance gene (*tetM*) that was amplified from pAS222. The P32 promoter was amplified from pMG36e. Primers used in this study are listed in Table 2. The *menF* and *menA* genes were cloned into the XbaI/HindIII sites of pMG36e using the appropriate restriction enzymes. The genes of the mevalonate and polyprenyl pathways, except *hmcM*, were cloned into the SmaI site of pMG36e by blunt-end cloning. Gibson assembly (Gibson et al., 2009) was employed to clone *hmcM* into the SmaI site of pMG36e, to clone *preA-menA* into pNZ8037 and for construction of pMEV-PP, pMEV-PP-2, and pCTR.

**Table 2:** Primers used in this study.

**Primers**	**Sequence 5^**′**^-3^**′**^**
**Construction of pMenF, pMenA, pHmcM, pThiL-MvaA, pMvk, pMvaD-Pmk, pFni, and pIspA:**	
Fw MenF	TATGTCTAGAGTTATAATTTCTATGGTAGAAAAAATG
Rev MenF	ATAGAAGCTTTCATAAGGCTTCTAAAATCGTTTTTAA
Fw MenA	TATGTCTAGAATCACATTAAAAGAGGAAATAATG
Rev MenA	ATAGAAGCTTTTAAAATCTAATCAAACTAATAAAGAGC
Fw HmcM ol pMG36e GC	AATTCGAGCTCGCCCATGAAAGTCGGTATTGATAAAC
Rev HmcM ol pMG36e GC	GAGGATCGATCCCCCAGAAAAGCTGTCAGTATTTTTTTATATTTTTTTATATTTAC
Fw pMG36e ol HmcM GC	AATTCGAGCTCGCCCATGAAAGTCGGTATTGATAAAC
Rev pMG36e ol HmcM GC	AGAGGATCGATCCCCCAGAAAAGCTGTCAGTATTTTTTTATATTTTTTTATATTTAC
Fw ThiL	CTTTCGGAGGTTCATTCGTGAAAG
Rev MvaA	TTATTTTCTCAAATTTTTTAGTAAATTTTGG
Fw Mvk	GCAGGAGAATTGTTAAAAATGAC
Rev Mvk	TTAAAAGGAGTAAATCCACGTG
Fw MvaD	TTTGATATAATAGTTTCATGAAAAATATTG
Rev Pmk	TCAGTTATTTTTTTGAGCAATCTTAAAC
Fw Fni	GAATTGAGAAATAAATGATGAAAAG
Rev Fni	TTATTTTTTTCTTTGTTGGATAAAATCG
Fw IspA	TGGTATAATTAGGGTAATGGATAC
Rev IspA	TTATTCCACTTCCAGTTGTTCAATT
**Construction of pMEV-PP, pMEV-PP-2, and pCTR:**	
Fw TetM ol P32 prom GC	CGTAATTCGAGCTCGCCCCGGCTTGTCTAGATTTGAATGG
Rev TetM ol pHH145 GC	GACTTTCTGCTATGGAGGTCAGGTATGATTTAAATGGTCACTAAGTTATTTTATTGAACATATATCGTAC
Fw P32 prom ol pHH145 GC	CAAAGTGATTAAATAGAATTCTCTAGATATCGCTCAATACATGGGTCGATCGAATTCG
Rev P32 prom	GGGCGAGCTCGAATTACG
Fw hmcM ol P32 prom	CGTAATTCGAGCTCGCCCGATAAGGAAATTTTTAAAATATGAAAGTC
Rev hmcM	TTATATTTTTTTATATTTACGATGGTTATCAAC
Fw thiL ol hmcM	GTTGATAACCATCGTAAATATAAAAAAATATAACTTTCGGAGGTTCATTCGTGAAAG
Rev mvaA	TTATTTTCTCAAATTTTTTAGTAAATTTTGG
Fw mvk ol mvaA	CCAAAATTTACTAAAAAATTTGAGAAAATAAGCAGGAGAATTGTTAAAAATGAC
Rev mvk ol preA GC	CTTTCCTCTCGATAATTAAAAGGAGTAAATCCACGTG
Fw preA ol mvk GC	ATTTACTCCTTTTAATTATCGAGAGGAAAGAGAAAAAC
Rev preA ol mvaD GC	AACTATTATATCAAACTTTTAATAATTTCGCTCTAATAAAATC
Fw mvaD ol preA GC	CGAAATTATTAAAAGTTTGATATAATAGTTTCATGAAAAATATTG
Rev pmk	TCAGTTATTTTTTTGAGCAATCTTAAAC
Fw fni ol pmk	GTTTAAGATTGCTCAAAAAAATAACTGAGAATTGAGAAATAAATGATGAAAAG
Rev fni	TTATTTTTTTCTTTGTTGGATAAAATCG
Fw ispA ol fni	CGATTTTATCCAACAAAGAAAAAAATAATGGTATAATTAGGGTAATGGATAC
Rev ispA	TTATTCCACTTCCAGTTGTTCAATT
Fw TetM ol ispA	AATTGAACAACTGGAAGTGGAATAACGGCTTGTCTAGATTTGAATGG
Rev TetM ol pHH145 GC	GACTTTCTGCTATGGAGGTCAGGTATGATTTAAATGGTCACTAAGTTATTTTATTGAACATATATCGTAC
Fw pHH145 GC	TGACCATTTAAATCATACCTGACC
Rev pHH145 GC	GTATTGAGCGATATCTAGAGAATTCTATTTAATC
Rev mvk ol mvaD GC	CATGAAACTATTATATCAAATTAAAAGGAGTAAATCCACGTG
Fw mvaD ol mvk GC	CGTGGATTTACTCCTTTTAATTTGATATAATAGTTTCATGAAAAATATTG
**Construction of pPreA, pGerCA-IspB and pPreA-MenA:**	
Fw preA BsaI	TTGAGGTCTCACATGCTCACATTTTGGCAGGATTATCCC
Rev preA XhoI	TGTCAACTCGAGCCTATCGGTGACAGGCTTTTAATAATTTC
Fw gerCA/ispB BsaI	TTGAGGTCTCACATGAATATCAAAGAATACGTTTATGTTTCCTTATTAAC
Rev gerCA/ispB XhoI	TGTCAACTCGAGAAAATTCATCAGGGGTCATAAAGTTCGCT
Fw preA menA GC	TACAAAATAAATTATAAGGAGGCACTCACCTTGCTCACATTTTGGCAG
Rev preA menA GC	TGCAGCCCGGGATCCATGTGCAGTACCCATTATTTAAAATCTAATCAAACTAATAAAGAG
Fw pNZ8037 GC	ATGGGTACTGCACATGGATC
Rev pNZ8037 GC	GGTGAGTGCCTCCTTATAATTTATTTTG

*ol, overlap; GC, primers used for amplification of fragments for Gibson cloning*.

HiFi DNA Assembly Master Mix (NEB, Ipswich, MA, USA) was employed for the Gibson assemblies. Overlap extension PCR (Horton et al., 1989) was used to combine the P32 promoter, *hmcM, thiL-mvaA*, and *mvk* into one fragment and *mvaD-pmk, fni, ispA*, and *tetM* to a second fragment before Gibson assembly with the *preA* and pHH145 fragments (4-component assembly). For pMEV-PP-2 a 3-component assembly was performed as described for pMEV-PP, but *preA* was left out of the reaction mix and primers were adjusted accordingly to amplify the mevalonate and polyprenyl pathway fragments. The tetracycline resistance gene (*tetM*) was positioned at the end, after all MEV and PP genes, to ensure transcription through the whole construct when transformed cells were cultured with tetracycline (pMEV-PP and pMEV-PP-2 Supplementary Figure 2). As a control, the *tetM* gene was assembled in a similar way into pHH145 behind the P32 promoter (pCTR, Supplementary Figure 2).

The *preA* and *gerCA-ispB* genes were cloned into the NcoI/XhoI site of pNZ8037 using appropriate restriction enzymes. Difficult fragments were subcloned into pCR™Blunt II-TOPO® vector (Invitrogen, St. Louis, MO, USA) and all inserts were routinely confirmed by DNA sequencing. Plasmids were transformed by electroporation into MG1363 or NZ9000 (Holo and Nes, 1989).

### Menaquinone Extraction and Analyses

The menaquinones of cells in culture were extracted essentially as described by others (Koivu-Tikkanen et al., 2000; Manoury et al., 2013) using a heptane:2-propanol mix (1:2, v/v) as extraction agent (2-propanol mix; 2-propanol:HCl (37%):MeOH 8.25:1:1 v/v/v). Phylloquinone (vitamin K1) at 40 ng/ml was included in the MeOH fraction and used as an internal standard. The extracts were analyzed by reverse phase HPLC on an UltiMate 3000 UHPLC system equipped with a Shiseido C18 (2.0 × 100 mm) column followed by a Shiseido CQ-R (2.0 × 20 mm) reduction column (Shiseido, Tokyo, Japan) and an RS FL fluorescence detector (Thermo Fisher Scientific, Rockford, IL, USA) set at 248 nm for emission and detection at 436 nm. The mobile phase was methanol:2-propanol (1:1, v/v), flow rate 200 μl/min, the injection volume was 0.5 μl and the column temperature 50°C. A sample containing standards MK-4, MK-7, MK-9, and K1 was employed for determination of retention times (Supplementary Figure 1A). The fluorescence response per mol was the same for all vitamin K standards. The molar concentrations of menaquinones were quantified using MK-7 as external standard (standard curve for MK-7 ranging from 10 to 1,000 ng/ml is shown in Supplementary Figure 1B). All reagents used for menaquinone extraction were of HPLC grade and standards K1 (95271), MK-4 (V-9378), and MK-7 (1381119) were purchased at Sigma (St. Louis, MO, USA). Standard MK-9 (M213610) was purchased at Toronto Research Chemicals (Toronto, ON, Canada). *P*-values were calculated using two-tailed *T*-tests.

## Introduction

Vitamin K is a family of essential, fat-soluble vitamins required for blood coagulation, but also involved in deposition and removal of calcium in various tissues (Flore et al., 2013; Schwalfenberg, 2017). The family comprises two naturally active vitamers: vitamin K1 (phylloquinone) produced by plants, and vitamin K2 (menaquinone, MK-n where n represents the number of isoprene units). Menaquinones are principally of bacterial origin, but MK-4 can be formed in mammals through conversion of phylloquinone (Okano et al., 2008). In humans and other mammals vitamin K is essential for its role as a cofactor for the enzyme γ-glutamyl carboxylase (Furie et al., 1999). This enzyme carboxylates glutamine residues on certain proteins into γ-carboxyglutamic acid (Gla) residues. Gla-proteins have increased affinity for calcium and are involved in protein-protein interactions (through Ca^2+^), cell membrane interactions and processes that promote correct deposition of calcium in bone and prevents deposition in soft tissues like arteries, cartilage, and heart valves (Wen et al., 2018).

A daily consumption of 0.75–1 μg vitamin K per kg body weight is regarded as the minimum adequate daily intake since it reinstates normal coagulation in elderly male patients with vitamin K deficiency (Frick et al., 1967). Life-threatening (primary) vitamin K deficiency, caused by excessive bleeding due to insufficient carboxylation of coagulation factors, is rare except in newborns (Vermeer, 2012; Schwalfenberg, 2017). The Western diet is thus sufficient to prevent acute disease. However, to fully carboxylate Gla-proteins other than the coagulation factors and thus prevent secondary (sub-clinical) vitamin K deficiency Western diets appear insufficient (Vermeer, 2012; Bruno, 2016). A long-lasting secondary vitamin K deficiency can lead to development of cardiovascular disease and osteoporosis (Szulc et al., 1993; Luukinen et al., 2000; Schurgers et al., 2005; Cranenburg et al., 2010; Shea et al., 2011; Vermeer, 2012; Schwalfenberg, 2017). Increased vitamin K intake appears to be beneficial and important for public health. The advantage of increasing the consumption of menaquinone compared to phylloquinone has been stressed significantly over the years. Firstly, menaquinone intake, but not phylloquinone has been shown to be inversely correlated to all-cause mortality, cardiovascular disease and certain cancers in large population-based studies (Geleijnse et al., 2004; Gast et al., 2009; Nimptsch et al., 2010). In addition, the longer menaquinones appear to have extended stability (days, compared to hours) and better bioavailability after ingestion compared to phylloquinone (Vermeer, 2012).

Our most important dietary sources of vitamin K2 are fermented foods like cheese and natto (fermented soybean). Fermentation of soybean by the bacterium *Bacillus subtilis* var. *natto* yield very high MK-7 amounts as levels up to 1,100 μg/100 g can be achieved (Schurgers and Vermeer, 2000). Regular consumption of natto could fulfill our requirement for vitamin K2, but unfortunately natto has a rather sharp taste and is not enjoyed much outside of Japan. In Europe and Northern America lactic acid bacteria (LAB) are the most important vitamin K2-producers for our diet as they ferment milk into dairy products such as cheese rich in vitamin K2 (up to 110 μg/100 g) (Manoury et al., 2013; Vermeer et al., 2018). LAB are highly valued and exploited in food fermentations and have potential to be used as cell factories for production of various metabolites for industry (Sauer et al., 2017). An extensive set of genetic tools has been developed for LABs over the years and this can enable efficient metabolic engineering of industrially important strains. Efforts have been made to enhance production of vitamins like riboflavin (Burgess et al., 2004; Chen et al., 2017; Juarez Del Valle et al., 2017), folate (Albuquerque et al., 2017; Saubade et al., 2017; Meucci et al., 2018) and cobalamin (Bhushan et al., 2017; Li et al., 2017) in LAB and thereby increase the functional value of fermented food, but until very recently there were no reports on optimization of dairy production or metabolic engineering of LAB strains to achieve higher menaquinone levels. Several genomes of the dominating vitamin K2 producing LAB, *L. lactis* have been sequenced, and putative genes encoding the enzymes for the individual steps of menaquinone biosynthesis annotated (Wegmann et al., 2007).

**Figure 1:**
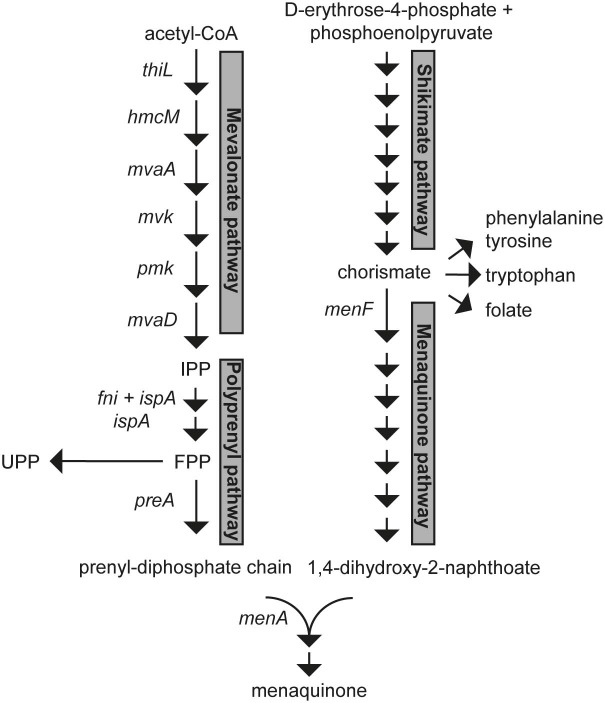
The lactococcal biosynthetic pathways for menaquinone(s). Intermediates that are substrates for competing essential pathways are shown as branched points (chorismate and FPP). IPP, isopentenyl pyrophosphate; FPP, farnesyl pyrophosphate; UPP, undecaprenyl pyrophosphate.

In *L. lactis* menaquinones are synthesized from acetyl-CoA, phosphoenolpyruvate and D-erythrose-4-phosphate. The precursors are converted step by step to a hydrophobic polyprenyl diphosphate (PP) chain (mevalonate and polyprenyl pathways) and a hydrophilic naphtoquinone ring; 1,4-dihydroxy-2-napthoate (DHNA) (shikimate and menaquinone pathways). Finally, MenA, a DHNA polyprenyltransferase, joins the prenyl diphosphate and DHNA to form demethylmenaquinone (Figure 1). The product of the shikimate pathway, chorismate, is also a substrate for synthesis of the essential aromatic amino acids (AAA) and folate and its production and further conversion is highly regulated (Dosselaere and Vanderleyden, 2001). For instance, the first enzyme of the shikimate pathway, 3-Deoxy-D-arabinoheptulosonate 7-phosphate synthase (DAHPS), is known to be feedback inhibited by AAA in diverse microorganisms (Mir et al., 2015) and similar regulatory events are likely to exist in lactococci. Chorismate is converted to DHNA through 7 enzymatic steps starting with isochorismate synthase (encoded by *menF*). The polyprenyl diphosphate chain is synthesized from isopentenyl pyrophosphate (IPP) units formed through the mevalonate pathway. Six enzymatic steps, catalyzed by 3-ketoacyl-CoA thiolase (*thiL*), hydroxymethylglutaryl-CoA synthase (*hmcM*), hydroxymethylglutaryl-CoA reductase (*mvaA*), mevalonate kinase (*mvk*), phosphomevalonate kinase (*pmk*), and diphosphomevalonate decarboxylase (*mvaD*) are required to convert acetyl-CoA into IPP (C5). These reactions constitute the most energy- and substrate-consuming part of menaquinone synthesis as they require 3 acetyl-CoA, 3 ATP, and 1 NADPH per IPP formed. The geranyltranstransferase (*ispA*) and isopentenyl-diphosphate delta-isomerase (*fni*) then combine IPP units into FPP (farnesyl diphosphate, C15). FPP is a scaffold for further lengthening of the prenyl diphosphate chain by consecutive addition of IPP units to make the all-trans polyprenyl diphosphate (for MK-n production) or di-trans, poly-cis-undecaprenyl pyrophosphate (UPP, C55). UPP is essential for lactococci as it is a substrate for synthesis of peptidoglycan (Bouhss et al., 2008). In *L. lactis* 2 genomic loci encode possible prenyl diphosphate synthases. The *gerCA* (*llmg_1111*) and *ispB* (*llmg_1110*) locus encodes proteins with homology to 2-component heptaprenyl diphosphate synthases. The gene *llmg_0196* is in an operon with *menA* (DHNA polyprenyltransferase) and encodes a putative geranylgeranyl pyrophosphate synthase. In *L. lactis* ssp. *lactis* the gene and gene product of *llmg_0196* are called *preA* and PreA and we will hereafter employ these names also for *L. lactis* ssp. *cremoris* MG1363.

Menaquinones play an essential role in electron transport but are not essential for fermentative growth in *L. lactis* (Rezaiki et al., 2008). However, in the presence of heme *L. lactis* can produce cytochrome and a functional electron transport chain enabling respiratory growth resulting in improved growth and survival in stationary phase (Sijpesteijn, 1970; Duwat et al., 2001; Gaudu et al., 2002; Rezaiki et al., 2008; Brooijmans et al., 2009). In addition, menaquinones can reduce both Fe and Cu and might be important for assimilation of metals (Rezaiki et al., 2008). The levels of menaquinone found in *L. lactis* are strain-dependent and vary in response to aerobic vs. anaerobic conditions as well as culture medium, carbon source and temperature. In *L. lactis* MK-9 is produced as the dominating menaquinone species, but minor amounts of MK-3, MK-7, MK-8 and MK-10 are also formed (Morishita et al., 1999; Rezaiki et al., 2008; Brooijmans et al., 2009). One recent study show that adjusting fermentation parameters like preculture conditions, carbon source and temperature result in up to 50% increase of vitamin K2 in fermented milk (Liu et al., 2019). However, there are no other reports available describing how to increase strain performance or optimize conditions to elevate menaquinone content during food fermentations, neither is information on the contribution of each enzyme to the biosynthetic pathway of menaquinones in lactococci. Such knowledge could be helpful in selecting the optimal lactococci for fermentation of milk into a product with higher functional value regarding vitamin K2. Therefore, in the present study several genes of the biosynthetic pathway of menaquinones in *L. lactis* ssp. *cremoris* MG1363 were overexpressed singly or in combination to investigate their potential to raise menaquinone levels. We identify bottle-necks and key genes for biosynthesis of menaquinones in MG1363 and thereby provide a foundation for development of strains capable of higher K2-production during food fermentations.

## Results

### Overexpression of *menF* or *menA* Increase Menaquinone Levels in *L. lactis*

**Figure 2:**
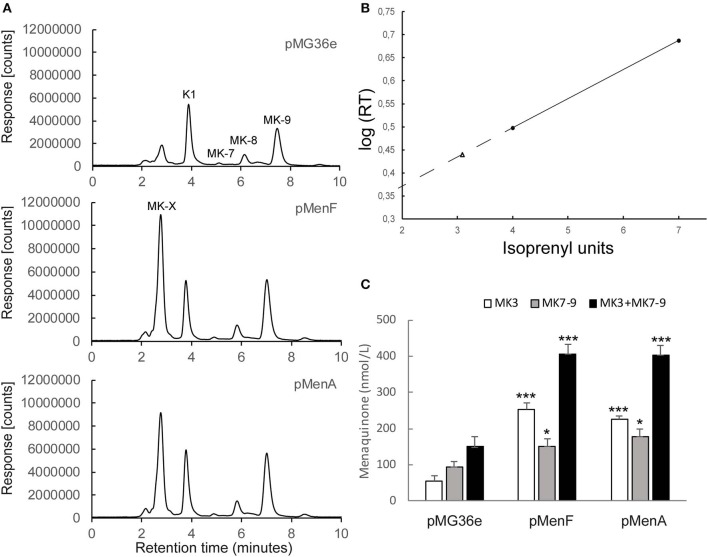
**(A)** Analytical HPLC chromatograms of quinones from recombinant *L. lactis* MG1363 cultures expressing additional lactococcal isochorismate synthase (encoded by *menF*) or DHNA polyprenyltransferase (encoded by *menA*) from P32 promoter of pMG36e. *L. lactis* containing empty vector (pMG36e) is shown in the upper panel for comparison. Peaks K1, MK-7 to MK-9 were identified based on retention times (RTs) compared to RTs of standards. **(B)** Plot of isoprenyl unit lengths vs. log_10_ (net RT in min) for standards MK-4 and MK-7. The linear function of the graph between 4 and 7 isoprenyl units was used to calculate the isoprenyl unit number of MK-X. The dashed line represents extrapolation of the graph. MK-X (RT = 2.75) is designated using a triangle and corresponds to 3 isoprenyl units (MK-3). **(C)** Quantification of MK levels from (a). Average and standard error of the means are shown from at least 3 independent experiments. The * and *** represent a *p*-value below 0.05 and 0.0005 respectively. The *p*-values were obtained using a two-tailed *T*-test where the strains overexpressing *menF* or *menA* were compared to the control strain carrying empty pMG36e.

We have employed “pull and push engineering” in our efforts to increase vitamin K2 production in MG163. Overexpression of isochorismate synthase (encoded by *menF)* was chosen in order to enhance flow through the shikimate pathway. More isochorismate synthase activity could create a metabolic pull through this pathway and increase flux into the menaquinone pathway (Figure 1). Overexpression of *menF* from the P32 promoter in pMG36e resulted in increased production (*p*-value 0.03) of the long-chained menaquinones MK-7, MK-8, and MK-9 (MK7-9) compared to the control strain (Figure 2A). The second gene chosen for overexpression, *menA*, encodes the DHNA polyprenyltransferase catalyzing the joining of the prenyl diphosphate chain and DHNA resulting in demethylated menaquinone. The content of MK7-9 in *L. lactis* expressing this gene from P32 on pMG36e was also higher (*p*-value 0.006) than the control strain (Figure 2A). Moreover, a dramatic increase of a short-chained menaquinone with retention time (RT) 2.75 min was observed (Figure 2A). A comparable increase in short chained MK content was seen for the *menF* overproducer. The presence of menaquinones with 3 prenyl units (MK-3) has been reported in lactococci (Rezaiki et al., 2008; Brooijmans et al., 2009). To determine the isoprenyl unit number of the short-chained menaquinone we employed the reported strategy by Rezaiki et al. (2008) and made a plot of the log_10_ (net RT in minutes) of standards MK-4 and MK-7 against their number of isoprenyl units (Figure 2B). The linear function of the graph was used to calculate the number of isoprenyl units for the short-chained menaquinone (MK-X). We determined the number of isoprenyl units for MK-X to be 3. From here onwards, we assume that the species with RT 2.75 min is MK-3.

The MK7-9 levels nearly doubled by overexpression of *menA* and increased by 60% when overexpressing *menF* (Figure 2C and Table 3). The concentrations of total menaquinones (MK-3 + MK7-9) in the cultures with the strain overexpressing *menF* or *menA* (Figure 2C) were both around 400 nmol/L on average, which is almost 3 times that of the control strain (140 nmol/L). Most of the increase was attributed to MK-3. The MK-3/MK-9 ratio was markedly lower for MG1363 (0.6) than the strains overexpressing *menF* (1.7) or *menA* (1.3) as seen in Table 3. We also observed that the ratio of MK-3/MK-9 in MG1363 could vary from 0.2 to 0.7 between experiments possibly reflecting growth medium variation.

**Table 3:** Summary of overexpression studies.

***L. lactis* host**	**Plasmid**	${\text{MK}}_{\text{Total}}^{*}$ (nmol/L)	MK7-9^*^ (nmol/L)	**MK-3/MK-9**
MG1363	pMG36e	150 ± 12.2	97 ± 25.4	0.6
MG1363	pMenF	406 ± 18.2	152 ± 27.3	1.7
MG1363	pMenA	404 ± 19.6	179 ± 26.1	1.3
MG1363	pHmcM	100 ± 26.6	81 ± 24.3	0.2
MG1363	pThiL-MvaA	218 ± 17.1	169 ± 22.4	0.3
MG1363	pMvk	314 ± 26.2	309 ± 23.6	<0.1
MG1363	pMvaD-Pmk	94 ± 14.8	88 ± 13.4	0.1
MG1363	pFni	100 ± 20.3	90 ± 22.5	0.1
MG1363	pIspA	61 ± 13.4	58 ± 13.8	0.1
MG1363	pCtr	172 ± 32.6	128 ± 28.1	0.3
MG1363	pMEV-PP	520 ± 33.7	364 ± 51.1	0.4
NZ9000	pNZ8037	143 ± 16.5	119 ± 16.3	0.2
NZ9000	pPreA	485 ± 28.7	467 ± 20.2	<0.1
NZ9000	pGerCA-IspB	66 ± 0.6	43 ± 4.0	0.5
NZ9000	pPreA-MenA	544 ± 75.1	544 ± 75.1	<0.1
NZ9000	pNZ8037 pMG36e	292 ± 3.4	238 ± 6.9	0.2
NZ9000	pPreA-MenA pMvk	719 ± 33.0	687 ± 35.6	<0.1
NZ9000	pNZ8037 pCtr	127 ± 1.5	114 ± 3.2	0.1
NZ9000	pMEV-PP-2 pPreA-MenA	657 ± 32.6	651 ± 30.2	<0.1

*Numbers for NZ9000 strains are from experiments using 2 ng/ml for induction of the nisA promoter*.

* *Average and standard error of the means of at least 3 experiments*.

### *llmg_0196* Encodes the Prenyl Diphosphate Synthase in *L. lactis*

Although we achieved a 3-fold increase in total MK production in *L. lactis* by overproducing either *menF* or *menA*, the isoprenoid chain length of the menaquinones produced was not optimal. Long-chained MKs are more desirable in foods than short-chained MKs since longer MKs have a longer half-life and stability in the blood and are also reported to have a stronger protective effect on the risk of coronary heart disease than shorter menaquinones (Gast et al., 2009; Sato et al., 2012; Vermeer, 2012; Bruno, 2016). There was considerably more MK-3 than MK7-9 made by the overproducers of MenF and MenA (Figure 2C). This indicates that DHNA is in surplus and the production of the isoprenoid chain appears to be limiting. We therefore reasoned that the high levels of MK-3 represents a potential for higher MK7-9 production and focused on enhancing the mevalonate and polyprenyl pathways to stimulate production of longer isoprenoid chains.

**Figure 3:**
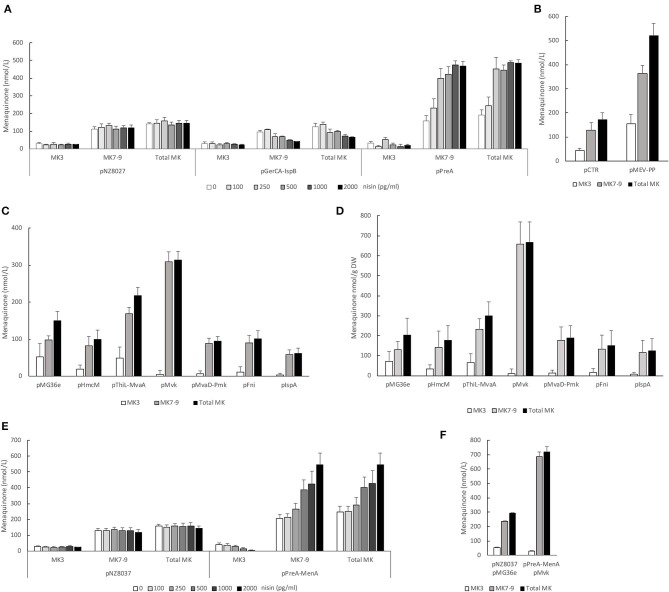
**(A)** PreA is a functional polyprenyl PP synthase in *L. lactis* ssp. *cremoris*. Strain NZ9000 containing empty pNZ8037 or expressing either *gerCA*+*ispB* or *preA* after induction of P_*nisA*_ using increasing concentrations of nisin. **(B)** Combined overexpression of all mevalonate and polyprenyl pathway genes in one transcript [genes were cloned in cis after the constitutive P32 promoter and inserted into pHH145 (pMEV-PP)]. Strain *L. lactis* ssp. *cremoris* MG1363 transformed with pMEV-PP or pCTR. **(C)** Overexpression of mevalonate and polyprenyl pathway genes from the P32 promoter of pMG36e. Strain *L. lactis* ssp. *cremoris* MG1363 transformed with pHmcM, pThiL-MvaA, pMvk, pMvaD-Pmk, pFni or pIspA. **(D)** Specific concentrations of vitamin K2 (nmol MK/g DW) when overexpressing mevalonate or polyprenyl pathway genes from the P32 promoter of pMG36e. **(E)** Combined overexpression of *preA* and *menA* and its effect on menaquinone production. Strain *L.lactis* NZ9000 containing empty pNZ8037 or transformed with pPreA-MenA after induction of P_*nisA*_ with increasing concentrations of nisin. **(F)** Combined overexpression of *preA, menA*, and *mvk* and its effect on menaquinone production. Strain *L. lactis* NZ9000 containing empty pNZ8037 and empty pMG36e or transformed with pMvk and pPreA-MenA. Induction of P_*nisA*_ with 2 ng/ml nisin. All strains were cultivated in GM17 and statically incubated at 30°C over night. Quantification of MK-3, MK7-9 and MK-3+MK7-9 levels from average of at least 3 independent experiments. Error bars represent standard error of the means.

To analyze the impact of mevalonate or polyprenyl pathway genes we first determined the involvement of the genes encoding the two putative prenyl diphosphate synthases; *gerCA*+*ispB* and *preA*. The coding sequences of either *gerCA*+*ispB* or *preA* was cloned into pNZ8037 and expressed using the inducible NICE expression system (de Ruyter et al., 1996) in strain NZ9000. Induction of the P_*nisA*_ promoter was regulated by addition of nisin at increasing concentrations (Figure 3A). We found that *preA*, but not *gerCA*+*ispB*, appear to encode a functional prenyl diphosphate synthase for MK production in *L. lactis*. When expression of *preA* was induced with nisin the levels of MK7-9 increased in a dose dependent manner reaching 480 nmol/L (Figure 3A and Table 3). This represented a fourfold increase in MK7-9 levels compared to control the strain pNZ8037. In contrast, menaquinone levels showed a slight, dose-dependedent decline upon induction of *gerCA*+*ispB* with nisin. There was no noticeable change in MK-3 levels as either prenyl diphosphate synthase was expressed.

### Increasing the Substrate Pool for Prenyl Diphosphate Synthase: Overexpression of Mevalonate and Polyprenyl (MEV-PP) Pathway Genes

Overexpression of *preA* was sufficient to increase MK levels close to 500 nmol/L. We reasoned that increasing the amounts of FPP and IPP, the substrates of PreA, could stimulate menaquinone production in the PreA strain even more. To this end we used Gibson cloning (Gibson et al., 2009) to construct a plasmid where all the genes of the MEV-PP pathways (Figure 1) could be expressed from the same promoter and possibly ensure an increased supply of IPP and FPP. The genes *hmcM, thiL, mvaA, mvk, preA, mvaD, pmk, fni, ispA* were cloned in pHH145 with the P32 promoter up front (Supplementary Figure 2). In *L. lactis* ssp. *cremoris* MG1363 transformed with pMEV-PP MK production increased 3-fold compared to MG1363 transformed with pCTR (from 172 to 520 nmol/L on average) (Figure 3B and Table 3).

In *L. lactis* total enzyme activity of the MEV and PP pathways is dependent on the amount of transcription from seven transcriptional start sites. It is likely that increased expression of all mevalonate and PP genes not necessarily infers the correct balance of each intermediate for optimal prenyl diphosphate chain production. To clarify this issue and determine whether any of the mevalonate or PP genes are more important for increasing the MK production than others they were overexpressed individually or in pairs (for genes where endogenous location is together in cis: *thiL*+*mvaA* and *mvaD*+*pmk*) from the P32 promoter on plasmid pMG36e (Figure 3C). Of all the genes overexpressed only *mvk* significantly increased total MK levels (*p*-value 0.002) which doubled compared to the control strain. The overexpression of *ispA* was the only gene that significantly reduced total MK levels compared to the control strain (*p*-value 0.02).

As several of these clones grew to lower cell density than the control strain, we included a measurement of the MK content on dry weight basis. As shown in Figure 3D most clones contained less MK-3/g DW than the control, while MK7-9 was the same or slightly elevated. However, overexpression of *mvk* led to more than 3-fold higher specific concentration of MK7-9, while MK-3 was reduced.

### Combined Overexpression of Key Genes

Increased expression of either *preA, menA, menF*, or *mvk* all led to at least doubled levels of MK and appear to be key genes for MK synthesis. Combined overexpression of several of these genes could have an additive effect on MK production, and this possibility was explored. As *preA* and *menA* are located after one another in an operon they were cloned together and overexpressed using the NICE expression system (Figure 3E). At maximal induction of cells transformed with pPreA-MenA we obtained an average concentration of 540 nmol/L MK7-9 (Table 3), the highest concentration obtained through our genetic engineering approach thus far. Next, we transformed this clone with pMvk (Figure 3F). In this strain the gene encoding mevalonate kinase is expressed constitutively and *preA* + *menA* upon induction with nisin. This led to another slight increase of MK production, reaching 680 nmol/L MK7-9 (Table 3). We note that the MK level in the control strain harboring the two empty plasmids is higher than what we have observed for other control strains. We also analyzed the MK production in a strain overexpressing all mevalonate and PP genes in addition to MenA (pMEV-PP-2 and pPreA-MenA). This strain produced an average of 651 nmol/L MK7-9 (Table 3), comparable to the strain overexpressing *mvk, preA*, and *menA*. All results obtained through overexpression studies are summarized in Table 3.

### Fermentation of Milk by Vitamin K2 Overproducing Strains

**Figure 4:**
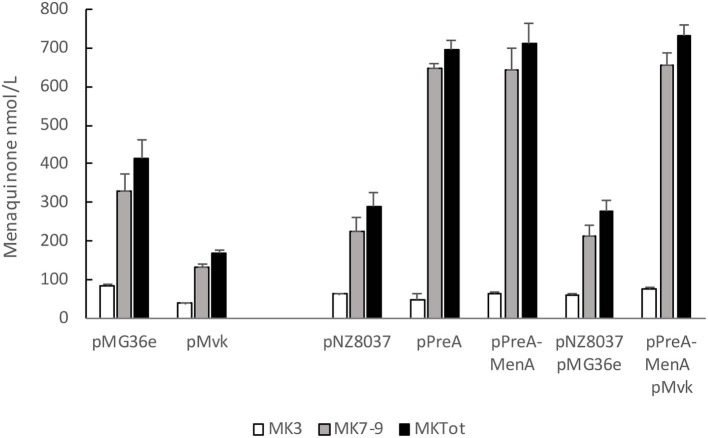
Vitamin K2 content in milk fermented by strains overexpressing key genes of the biosynthetic menaquinone pathway. Fermentation was carried out for 20 h at 30°C in heat-sterilized skimmed milk supplemented with 0.5% glucose and 1% tryptone. Nisin (2 ng/ml) was added to NZ9000 strains 1 h after inoculation. Quantification of MK-3, MK7-9 and MK-3+MK7-9 levels from average of at least 3 independent experiments. Error bars represent standard error of the means.

Finally, we tested whether overexpression of the key genes identified earlier affect MK content also during fermentation of milk (Figure 4). All strains except the overproducer of mevalonate kinase acidified the milk to a pH below 5.0 and caused coagulation, and this strain also produced less MK than the control strain in milk. Overexpression of *preA* alone or together with *menA* or *menA*+*mvk* caused elevated production of vitamin K2. The total vitamin K2 levels reached close to 700 nmol/L (500 ng/g fermented milk) using any of the 3 strains, and MK7-9 constituted more than 93% of the total menaquinone content.

## Discussion

Subclinical vitamin K deficiency, not uncommon in the Western world, is associated with increased risks of diseases including osteoporosis and cardiovascular disease (Geleijnse et al., 2004; Gast et al., 2009; Schwalfenberg, 2017). Being more effective than vitamin K1, increasing the intake of long-chained vitamin K2 would be the best way to improve vitamin K status. In this study, we have analyzed MK biosynthesis in the most important menaquinone-producer for our diets; *L. lactis*. Our results demonstrate that it is possible to increase MK production by *L. lactis* by enhancing transcription of key genes like *menF, menA, preA*, or *mvk*.

Metabolic pathway engineering for increased product formation often involve increasing the substrate pools. Chorismate represents a critical branch-point in menaquinone biosynthesis since it is also a substrate for production of the essential AAA and folate. A good strategic starting point for metabolic engineering of *L. lactis* could be to ensure increased chorismate levels and thereby elevate the supply of substrate for isochorismate synthase. However, efforts to increase chorismate levels by manipulating the shikimate pathway have proven unsuccessful in *B. subtilis*, probably due to increased feedback inhibition of the shikimate pathway caused by a concomitant rise in AAA levels (Tsukamoto et al., 2001; Yang et al., 2019). Moreover, by overexpressing enzymes involved in conversion of chorismate to folate Wegkamp et al. (2007) obtained considerable increases in folate production in *L. lactis*. Basic levels of folate production were in the same range as MK production, indicating that flux through the shikimate pathway would not be a bottleneck in our work. Our first targeted genetic approach was consequently aimed at steps outside of the shikimate pathway by trying to push carbon flux into the menaquinone pathway or pull flux through both pathways by cloning and expression of *menF* and *menA*, respectively. Overexpression of either *menF* or *menA* increased total menaquinone levels and MK-3 was the main menaquinone generated. The increase in MK-3 levels can be explained by a shortage of long-chained polyprenyl diphosphates compared to the amount of DHNA present and pinpoint prenyl diphosphate chain synthesis as rate-limiting for MK synthesis. As DHNA prenyltransferases are highly specific for DHNA, but unspecific for the prenyl diphosphate chain, MenA will join DHNA with any prenyl diphosphate chain available (Saito and Ogura, 1981). Therefore, in the overproducers of MenA and MenF FPP (C15) appears to be in excess as MK-3 is formed in large quanta. Longer prenyl diphosphate chains (C40-C50) form by sequential condensation of FPP with 5-7 IPP units in a reaction catalyzed by prenyl diphosphate synthase (Ogura and Koyama, 1998; Koyama, 1999). The mevalonate pathway, which is required for formation of IPP (C5), or prenyl diphosphate synthase activity consequently appear limiting in the MenF and MenA overproducers. MK-3 can be a major contributor to the total menaquinone content in lactococci (Brooijmans et al., 2009) in agreement with our data. The presence of MK-3 indicate that the mevalonate and polyprenyl pathways are also limiting in the wild type. Cells overexpressing PreA produced long chained MK instead of MK3 showing that prenyl diphosphate chain elongation activity and not IPP supply limits MK synthesis. MK-3 was reduced when the mevalonate or polyprenyl pathway genes were overexpressed, likely to be caused by the metabolic pull created by the increased enzymatic activity.

In late growth energy supply is reduced and the production of MK-3 relative to MK-9 has been shown to increase in *L. lactis* (Rezaiki et al., 2008). Interestingly, *menF* transcription was found to increase by aerobic conditions (Cretenet et al., 2014), but the MK-3/MK-9 ratio decreased (Brooijmans et al., 2009). Aeration alters metabolism, and improves energy and redox status in *L. lactis*, and this may favor enhanced synthesis of IPP.

From the *menF* and *menA* overexpression studies it is apparent that there is a lack of prenyl diphosphate synthase activity to create longer isoprenoid chains so that the more valuable longer menaquinones can form. The genome of *L. lactis* ssp. *cremoris* MG1363 contains ORFs encoding 2 different putative prenyl diphosphate synthetases. When overexpressing *llmg_0196* (*preA*) MK7-9 production increased 3-fold. The length of the isoprenoid side chains of menaquinones is defined by the prenyl diphosphate synthases and these enzymes are classified as short-, medium-, or long-chained prenyl diphosphate synthases accordingly (Ogura and Koyama, 1998; Koyama, 1999). Long-chain prenyl diphosphate synthases are homodimers that add IPP units to allylic diphosphates generating C40 and longer prenyl diphosphate chains. Our results indicate that PreA is a functional long-chain (C45) prenyl diphosphate synthase as the levels of longer menaquinones are increased when transcription of *preA* is induced.

The second putative prenyl diphosphate synthase of MG1363 is encoded by 2 overlapping ORFs annotated as *gerCA* and *ispB*. Heterodimeric prenyl diphosphate synthases generate medium chain prenyl diphosphates (C30 and C35) (Ogura and Koyama, 1998; Koyama, 1999). However, we did not observe an increase in the amount of medium length MKs when overexpressing *gerCA*+*ispB*. This implies that the *gerCA*+*ispB* locus could either contain a non-functional prenyl diphosphate synthase or that GerCA+IspB generates a polyprenyl diphosphate chain used for something different than MK production. Since MK7-9 levels were reduced by increased expression of *gerCA*+*ispB* we assume that less substrate (FPP, IPP) became available to maintain normal PreA activity and MK production.

Mevalonate kinase was identified as a key gene for MK production in MG1363 as overexpression of *mvk* doubled the amount of MK produced. This result is in accordance with Song et al. (2014) who identified *mvk* to be a metabolic bottleneck of the mevalonate pathway. Overexpression of other mevalonate genes did not result in greatly increased MK levels. We observed that overexpression of several mevalonate or polyprenyl pathway genes, especially *mvk*, inhibited growth as seen by the lower OD600 reached after overnight cultivation. Alterations of the mevalonate or polyprenyl pathway enzyme activity could result in a build-up of mevalonic or isoprenoid intermediates. An abundance of isoprenoid precursors is cytotoxic (Martin et al., 2003; Sivy et al., 2011), likely true also for *L. lactis*. When *mvk* was overexpressed together with *preA* and *menA* the growth defect was abrogated. In this strain increased mevalonate kinase activity is accompanied with increased PreA and MenA activity withdrawing possible toxic isoprenoid precursors.

To increase menaquinone production by bacteria several approaches has been explored. They include optimization of the fermentation process, increasing menaquinone secretion, improving bioreactor design, directed mutagenesis of strains or genetic engineering of strains (Ren et al., 2019). To enhance strain performance genomic changes has been made in order to maximize substrate pools, limit the production of by-products, overexpress key genes, express novel pathways, or combinations of the aforementioned strategies (Kong and Lee, 2011; Liu et al., 2017, 2018; Xu et al., 2017; Ma et al., 2019; Yang et al., 2019). Most of these studies have been performed using *B*. *subtilis, B. amyloliquefaciens, E. meningoseptica*, or *E. coli* and seek to produce menaquinone on an industrial scale for use as a nutritional supplement or food additive. The present study is the first to genetically engineer menaquinone synthesis in *L. lactis* and in a proof-of-principle manner use these GMO strains for vitamin K2 fortification of milk. We employed several of the strategies mentioned above to increase menaquinone production: limiting the production of by-products by committing substrates to menaquinone production (*menF, preA*), identification and overexpression of key genes (*menA, preA, mvk*) and increasing substrate pools (pMEV-PP). Based on our results and others there appear to be some general approaches that are successful when aiming to overproduce MK among diverse bacteria including *L. lactis* ssp. *cremoris*. Firstly, enhanced expression of *menA* result in around 2-fold higher MK production in *E. coli, B. amyloliquefaciens, E. meningoseptica*, and *B.subtilis* (Kong and Lee, 2011; Liu et al., 2017, 2018; Xu et al., 2017; Ma et al., 2019; Yang et al., 2019) and we found that this also applies for *L. lactis*. More MenA activity will pull out products of both the menaquinone and MEV+PP pathways possibly leading to an increased flux through both pathways. Secondly, optimizing the precursor pool for prenyl diphosphate synthase appears to be a fruitful strategy to increase MK production. In most bacterial species IPP form through the mevalonate-independent pathway called the MEP pathway (Frank and Groll, 2017). Overexpression of MEP and/or PP pathway genes increase MK production in *B. subtilis, E. coli* and *E. meningoseptica* (Kong and Lee, 2011; Ma et al., 2019; Yang et al., 2019). In line with these studies we achieved a doubling of the menaquinone content by overexpression of the *mvk* gene of the IPP-producing mevalonate pathway in *L. lactis*.

By cloning all genes of the MEV and PP pathways and expressing them from the P32 promoter we expected to increase MK production compared to single expression of *mvk* or *preA*. However, we achieved only slightly higher MK levels than by overexpressing *preA* alone using the NICE system. The difference in promoter strength between the constitutive P32 and the strong P_*nisA*_, might be a reason why combined expression of all MEV+PP genes did not evoke a higher MK production than *preA* alone.

We achieved a maximum MK7-9 titer of 687 nmol/L when combining overexpression of *menA, preA* and *mvk*. An additive effect on MK production when increasing the precursor pool for the prenyl diphosphate synthase combined with increased MenA activity has also been reported by others. In *E. menigoseptica* a 2.5-fold increase was achieved by combining overexpression of *menA* and a single MEP pathway gene (Liu et al., 2018). In *B. subtilis* a recent study reported 11-fold increase by overexpressing *menA* and three genes of the MEP pathway (Ma et al., 2019).

Five times increased MK production has been achieved by overexpression in *E. coli* and *B. subtilis* (Kong and Lee, 2011; Yang et al., 2019) similar to the fourfold increase reported here. Except from the clones overexpressing single mevalonate genes there was no change in growth yield in *L. lactis* overproducing vitamin K2. The highest specific vitamin K2 content was 0.67mg/g DW, slightly lower than the 290 μg MK/g wet cell weight (=1.26 mg/m DCW) (Glazyrina et al., 2010) in the *E. coli* overproducer (Kong and Lee, 2011). However, the specific menaquinone content in *B. subtilis* can get much higher, and Yang et al. (2019) reported 12.0 mg/g DCW after optimization of culture conditions of their overproducing clone.

As a proof-of-principle, we employed several of the MK overproducing strains to verify whether these strains would increase menaquinone content of fermented milk to a beneficial level. Fermented milk is a dairy product mostly consumed in Nordic countries and contains vitamin K2 levels up to 80 ng/g (Koivu-Tikkanen et al., 2000; Liu et al., 2019). We achieved a significantly higher level using our overproducers as we obtained more than 5 times higher MK7-9 levels (450 ng/g). We also found that MK levels in general were higher after milk fermentation than by culturing in M17. The daily requirement for vitamin K is set at 1 μg/kg body weight (Frick et al., 1967). A serving of 200 ml fermented milk produced by our genetically engineered menaquinone overproducers would contain 90 μg long chained vitamin K2 and fulfill the daily requirement for most people. Hard cheese contains an average of 30–40 μg MK/100 g (Manoury et al., 2013; Vermeer et al., 2018). The average pro capita cheese consumption in Western countries is in the range of 41–82 g cheese/day (IDF, 2016) corresponding to a daily vitamin K2 intake of 12–32 μg. In this work we have shown by cloning that the bacterial specific vitamin K2 content can be increased fourfold, suggesting that a similar stimulation can be achieved in a cheese. With a vitamin K2 content of 120–160 μg MK/100 g such cheese could be a main contributor to meet the daily requirement of vitamin K even in people with a moderate cheese intake.

We have pinpointed the mevalonate and polyprenyl pathways as rate-limiting for MK synthesis and increasing the pool size of the precursor acetyl-CoA could be a strategy for further improvement of vitamin K2 production. Possibly this could be achieved by redirecting carbon flow from homolactic to more mixed acid fermentation. However, increasing flux from pyruvate to acetyl-CoA would also affect the taste of the dairy product in a negative manner (Gaspar et al., 2011).

## Conclusion

Biosynthesis of menaquinones require over 20 enzymatic reactions and it is reasonable to expect that an elevated level of a single enzyme is insufficient to dramatically increase the amount of pathway product. However, when it comes to vitamin K2, just doubling or tripling the amount in our food could play a vital role for public health. Here, we have shown that overexpression of key genes is enough to double (*mvk*), triple (*menA*) and even quadruple (*preA, preA* + *menA*) vitamin K2 levels produced by the important vitamin K2 producer *L. lactis* ssp. *cremoris* MG1363 under laboratory conditions. We further demonstrate how these strains can ferment milk and increase the vitamin K2 content 3-fold in the resulting fermented milk. A minimal step to achieve 3 times higher levels of the long-chained MKs could therefore be to modify the endogenous promoter of the *preA*-*menA* operon to enhance transcription. The use of genetically-modified organisms (GMOs) for food production is under heavy jurisdiction in most countries, nevertheless over 100 GMOs are approved worldwide for use in commercial food or feed so far (Kamle et al., 2017). The rise of CRISPR-technology, to make precise genetic alterations in organisms ranging from bacteria like *L. lactis* (Guo et al., 2019) to human beings, is also believed to impact the legislation around GMO's and food production possibly enabling the use of such GMO's in a near future.

## Data Availability Statement

The datasets generated for this study are available on request to the corresponding author.

## Author Contributions

CB performed all the experiments except construction of pHH145 which was carried out by HH. CB and HH designed, analyzed, and interpreted all experiments and wrote the paper.

### Conflict of Interest

The authors declare that the research was conducted in the absence of any commercial or financial relationships that could be construed as a potential conflict of interest.
