# Supplementary figures and images for: Engineering Lactococcus lactis for Increased Vitamin K2 Production
Source: Front Bioeng Biotechnol. 2020 Mar 18;8:191. doi: 10.3389/fbioe.2020.00191 (PMC7093718; doi:10.3389/fbioe.2020.00191)

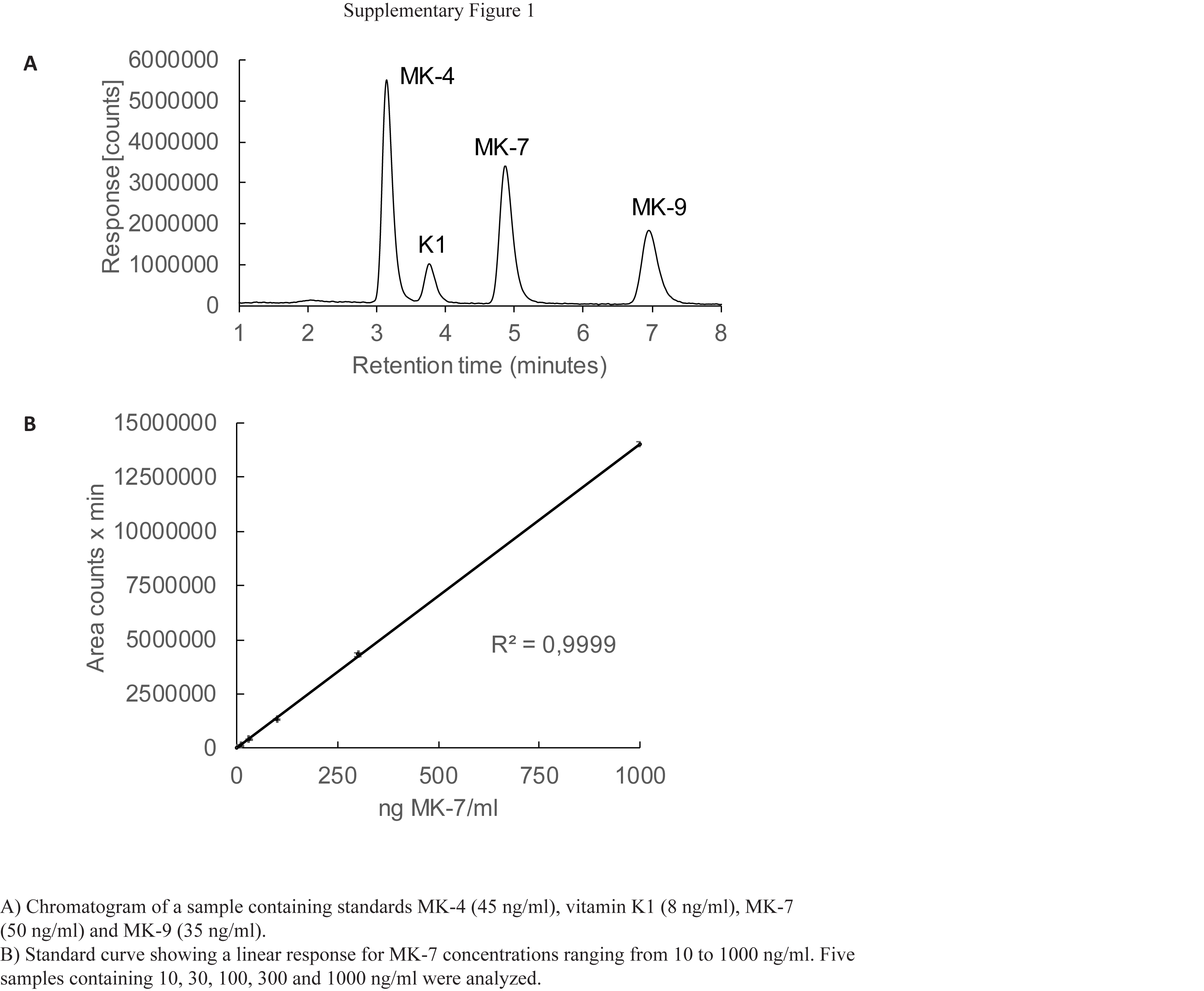

Supplement: Supplementary file 1 [file Image_1.TIF]

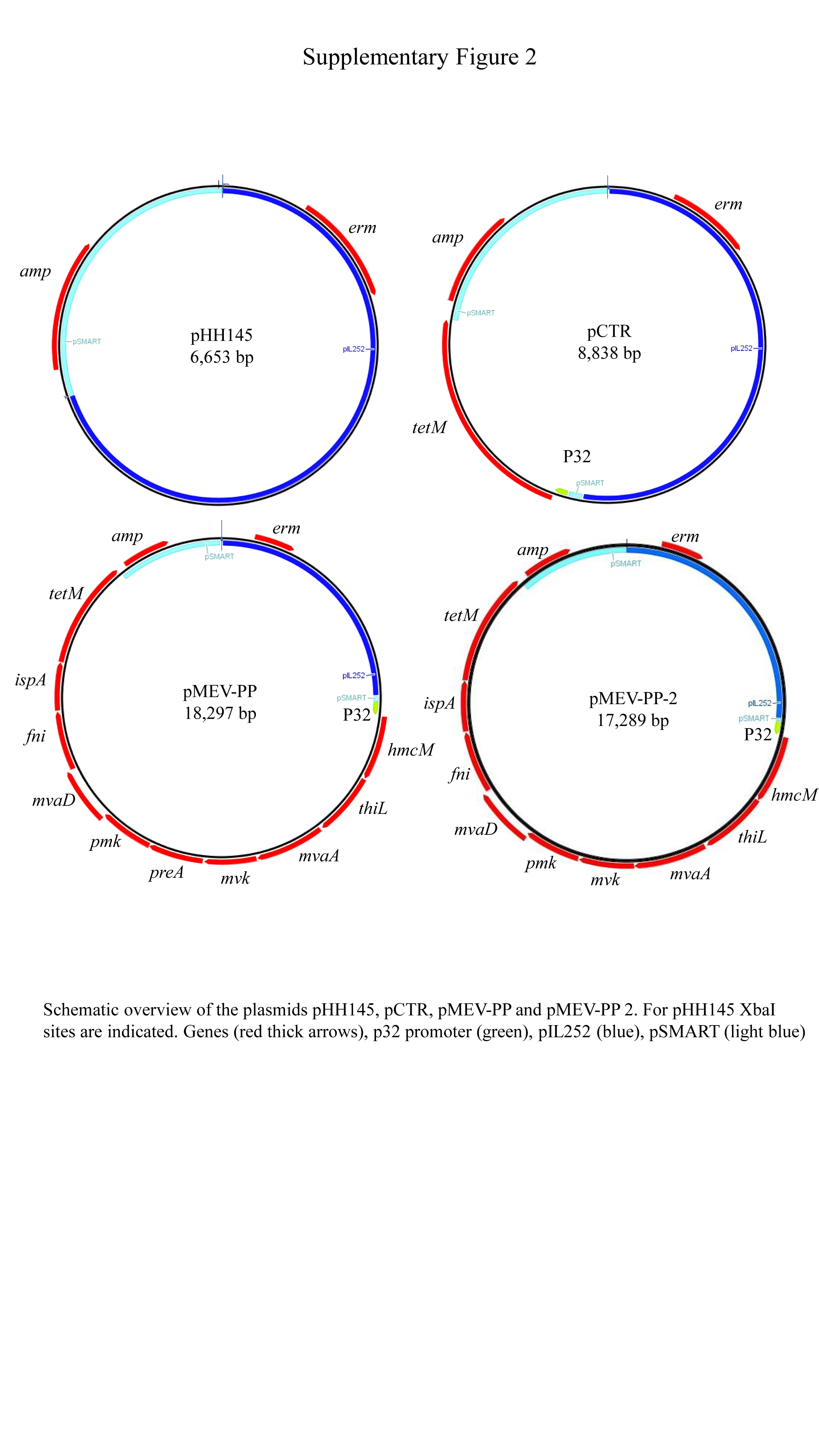

Supplement: Supplementary file 2 [file Image_2.TIF]
